# Modeling and scientific analysis of pediatric medication evaluation based on MDM-DEA-Malmquist model: construction of health management in pediatrics in developing countries

**DOI:** 10.1186/s13052-025-01893-0

**Published:** 2025-02-07

**Authors:** Kaixian Fang, Shaoqin Xue

**Affiliations:** Huzhou Maternity & Child Health Care Hospital, Huzhu, Zhejiang 313000 China

**Keywords:** Pediatric Medication, MDM Matrix, Lean Model, Assessment, DEA-Malmquist, Health Management

## Abstract

**Background:**

The developing countries represented by China have huge population potential and child population. However, due to economic and social development constraints, pediatrics need help regarding resources and technology. The level of pediatric medical care remains inadequate.

**Methods:**

This article constructs an MDM matrix framework for the pediatric medical nursing level. Combining the lean model theory, the key indicators of children's medical care are analyzed. Thus, it helps medical institutions scientifically evaluate and guide pediatric nursing. Based on the linkage extension of the MDM model and the DEA-Malmquist model, an evaluation system was established to reflect the current situation of pediatric drug use in China. The study used provincial indicator data on pediatric medication in China from 2015 to 2021.

**Results:**

We found that indicators such as physician literacy, health records, child status, and parental awareness were the most important under different medication cycles and goals. The importance of pre-administration evaluation was as high as 53.5%, and the importance of post-administration evaluation was only 13.7%. The efficacy of pediatric medication in developed provinces was significantly optimized. The input–output structure of pediatric medication in superior provinces was consistent with the feedback sequence of the MDM matrix.

**Conclusions:**

This indicates that the evaluation results of the MDM matrix have guiding significance for optimizing pediatric medication efficacy. This paper aims to help developing countries establish an optimized pediatric medication evaluation system and improve the pediatric healthcare environment.

**Supplementary Information:**

The online version contains supplementary material available at 10.1186/s13052-025-01893-0.

## Background

For a long time, developing countries have been facing problems such as resource constraints and difficulties in development. Their economy, technology, and living standards are significantly lower than developed countries. However, the population size of developing countries is as high as 6.39 billion. Their land area and population volume account for more than 70% of the world. Developing countries have become major production bases for agriculture, industry, and economic activities in the world[ [1]. The proportion of newborns born in developing countries is nearly 80% of the annual global population of about 78 million [2]. Against the backdrop of a gradually negative birth rate in developed countries, developing countries have colossal population potential and provide the world with substantial labor resources. Unfortunately, developing countries have difficulty creating a practical population value and even face a huge population burden. Developed countries have transferred important population resources, creating a siphoning effect in developed countries and further compressing the space for developing countries to build. Developing countries have been completely reduced to working as laborers and are gradually locked into low-end economic activities [3].

Resources for child care in developing countries are more vulnerable due to infrastructural, economic, human, social, and cultural weaknesses. Their pediatric systems and pediatric healthcare activities are fragile. Taking China, a developing country, as an example. Even though China is close to the level of developed countries in all areas and represents a higher level of developing countries. However, China's pediatric construction still needs to be improved. As of 2022, the total number of children aged 0–14 in China will be about 230 million, accounting for 18% of the country's total population [4]. Moreover, there are only 118,000 professionals qualified as pediatric occupational physicians. Only 0.53 professional pediatric care is available for every 1,000 children [5]. It takes about 1,800 children to receive care from one pediatric professional. It is important to realize that this is only for essential pediatric personnel [6]. The number of pediatric professionals with advanced titles or high levels of expertise is far lower. In addition, there is a mismatch and imbalance in the resources of pediatric medical institutions in China. In China, 43.6% of pediatric outpatient services and 53.5% of pediatric emergency services are provided by general hospitals. The proportion of specialized pediatric hospitals accounts for only 0.5% of local pediatric medical services. In rural areas, this figure is almost 0. This means that China needs more specialized or well-targeted pediatric health care. Practical, standardized, and effective medication activities in the pediatric healthcare system are the key landing points for ensuring the efficiency of pediatric healthcare and promoting the healthy growth of minors. The Declaration of Alma-Ata states that the principles of self-participation and State medical care are central to promoting a standardized basic pediatric health care system. Moreover, the accessibility of public health and essential healthcare services determines the height of children's growth [7]. The public will be guaranteed access to high-quality, efficient, affordable medical services. The establishment of a composite health insurance payment mechanism and a multi-level medical security system based on clinical pathways. The goal of synergistic development with a high-quality and efficient health care system; and reforms and tilts around children's medication have become the focus of pediatric construction in the next phase [8].

On a practical level, China's pediatric medicine is also facing a tight situation. Compared with developed countries, the medical foundation of developing countries could be more robust. There is an extreme need for medical professionals, and their R&D laboratories, R&D systems, and experimental activities for innovative medicines are all significantly underdeveloped [9]. This means that developing countries rely on imported pediatric drugs or generic drugs. Limited by the domestic economic level, even after the introduction of the production of children's drugs, the level, effect, and price are less ideal than in developed countries. Data show that China's number of children's medical consultations is increasing by 5 million annually. In 2021, the pediatric drug market reached 107.9 billion yuan, with a CAGR of 9.9%. Regarding incremental data on pediatric drugs, as of May 2022, the number of approved pharmaceutical products in China was about 18,400. However, 95% of the drugs were non-pediatric, and there were only 930 pediatric drugs. Regarding pediatric drug stock data, 90% of pediatric drugs have only 1 product specification and rely heavily on imports or single manufacturers. Only 6% of products have more than two specifications, and only 3% of pediatric products have more than three specifications. In addition, China's pediatric drugs are dominated by granules, tablets, and oral solutions, accounting for 32%, 25%, and 21%, respectively, in 2021 [10]. The combined share of the three dosage forms reaches 78%. Dispersions, pills, and capsules are relatively few, accounting for less than 10%. Regarding indications, China's pediatric drugs are dominated by general drugs for the respiratory system, anti-infective drugs, and the digestive system. The share in 2021 reaches 38%, 23%, and 17%, respectively, with a severe lack of the remaining specialty or targeted drugs. For other developing countries, this figure may be even more unsatisfactory. In addition, at a deeper level, there are endogenous problems with pediatric drug use. Children's medicines do not taste good, and medication compliance is poor. For a long time, there have been situations such as "use medicine by breaking, dose by guessing" and "pediatric discretionary reduction." The abuse of drugs by children has seriously harmed children's health. At the same time, China's pediatric medication drug development started late, and technology research and development are relatively backward. The number of approved pediatric products and active ingredients is relatively small, and the corresponding number of approved products accounts for less than 2%. Compared with China's 250 million children population, the contrast is enormous. At a high level, China's pediatric medication has both structural conflicts of insufficient supply and demand, as well as overflow problems of low-end drug reuse. There exists both the rigid problem of insufficient doctor-patient communication mechanisms and the soft problem of overly controlling parents and inadequate nursing literacy. There is both a lack of scientific and standardized medication strategies and a confused medication orientation that emphasizes inputs and results.

Developing countries account for most of the world's population resources and constitute a significant force in population turnover. Developing countries have the largest population of children in the world [11]. However, there is a severe shortage of resources for pediatric specialists and pediatric medicines. This not only restricts the construction of developing countries but also limits the quality of development of the world [12]. Overall, scientific management of pediatric medication has become an urgent issue for developing countries. According to statistics, approximately 30000 children in China suffer from hearing loss, and around 7000 children die each year due to improper medication. The incidence of adverse drug reactions in children in China is 12.5%, which is twice that of adults and four times that of newborns. The drug damage caused by irrational and incorrect use of drugs in children is more serious [13]. In the child population, drug poisoning accounted for 53% of all poisoned children seeking medical treatment in 2012 and increased to 73% in 2014. In terms of the age of poisoning, children aged 1 to 4 years old account for the most significant proportion of drug poisoning among children aged 0 to 14 years old. The problem of pediatric medication occurs in multiple stages before, during, and after medication. In the early stage of medication, there are still problems, such as a shortage of medication varieties, inappropriate dosage forms, and specifications for children [14]. The insufficient infrastructure capacity of hospitals and assessing children's condition have weakened medication effectiveness. In the middle and late stages of medication, 84.9% of children have safety hazards with medication, and most parents lack awareness of safe medication. Unreasonable or even incorrect medication is frequently used. Meanwhile, due to inadequate community management, there is a lack of personalized dispensing services for children's medication [15]. There is also a lack of an individualized precision medication system. From a profound background perspective, we need a scientific pediatric medication evaluation system to help pharmaceutical managers better carry out pediatric healthcare. For countries such as China, lean management of pediatric medication activities with limited resources can significantly improve the efficiency of pediatric medication. At the same time, it also prevents more children from facing problems such as resource loss and medication injuries. It also helps hospital managers reduce diagnosis time and improve work efficiency [16–18]. This provides the necessary research background for this study: breaking through resource constraints and constructing a lean evaluation system for pediatric medication. Therefore, the immediate problem is realizing the precise pediatric medication in developing countries under the limited resource environment and realistic conditions [19]. Considering multiple human, social, and economic constraints, how can we achieve efficient pediatric medication use in developing countries in the context of insufficient pharmaceutical resources? This paper aims to construct a pediatric medication assessment framework with characteristics of developing countries, help doctors and parents in developing countries have a better knowledge of pediatric medication, and select specific medications in a targeted manner [20]. Due to the significant variability within developing countries, this paper mainly selects empirical data from China. However, the pediatric assessment framework provided in this paper is strongly compatible with the MDM matrix system (Multiple-Domain-Matrix). Other scholars can improve the pediatric medication assessment system and provide ideas for pediatric health care in their countries based on real-life factors. Based on the assessment results of the MDM Matrix, this paper further constructs an input–output matching model for pediatric medication. With the help of MDM matrix thinking and assessment effects, this paper provides theoretical guidance and realistic feedback on the input–output system. This paper then uses the DEA-Malmquist model to provide feedback on the specific efficiency of pediatric medication in each province in China. Thus, the MDM matrix assessment idea is validated, and scientific strategies for pediatric medication use are given. Another significant contribution of this paper is that we do not call for the rapid establishment of pharmaceutical R&D and management systems in developing countries (which is a long transition process and requires sustained efforts by multiple generations of governments in the economic, social, educational, and innovation fields), considering the urgent problems of reality. Instead, we hope to sift through the limited factor environment to identify important pediatric medication assessment ideas that can quickly and qualitatively help children access excellent welfare benefits.

Pediatric medication has long been the focus of pediatric theory and practice. Based on CiteSpace, Li combed the literature studies on pediatric medication use in China and other countries. He found that Chinese pediatrics have continued to focus on directions such as rational and safe medication use, while foreign literature emphasizes the dimensions of medication diagnosis, safety assessment, and innovative research and development [21]. The focus of this study was to identify areas of action in pediatric medication use and to summarize the key motivations that influence pediatric medication use. Such motivations clearly vary between countries. In developing countries, most studies focus on surveys of over-the-counter medication use in hospital outpatient clinics. This highlights a dimension of pediatric development in developing countries that focuses on the outcome orientation of medication use [22]. On the one hand, they need to screen for cost-effective drugs with limited drug resources. On the other hand, due to the lack of drug safety or evaluation trials, they can only invest more effort at the clinical level of drugs [23]. In developed countries, most of the research is focused on the pediatric drug development level. They encourage the development of drug research and development work on child-specific dosage forms and specifications, and improve the specification of children's clinical use of drugs. Therefore, from the existing level, this motivation is manifested in developing countries in the form of multidimensional safety assessment of pediatric medications. In developed countries, they focus more on the cutting edge and innovation of pediatric medication. That is, developing or experimenting or applying pediatric medication drug with maximum efficacy within the maximum boundaries of safety [24].

In addition, limited by the economic level, developing countries also show the characteristics of pursuing short-term time-saving and low price in pediatric medication. Based on the analysis of big data of pediatric outpatient prescriptions in China, We found that the irrational rate of pediatric medication use reaches 15.02%. Among them, the misuse of hormones or antibiotics dominates the problem. Due to the gap between national conditions and the popularity of medical education, parents in developing countries are slightly less cognizant, which leads to the eagerness of doctors to use medication. In order to provide quick relief to the child and soothe the parents' anxiety, doctors often use heavy medications [25]. In addition, in response to outpatient emergency room turnover rates, as well as to larger waves of influenza and pediatric patients, more pediatric patients can often be cared for with more generalized and abbreviated medications. Therefore, antibiotics and hormones, which are better generalized, have become the mainstream means of medication administration. Among the mainstream pediatric drug resources, most of the hormones and antibiotics are relatively inexpensive, which meets the requirement of most families for inexpensive drugs. Moreover, the effect of medication in providing quick relief is generally recognized by the market. In developed countries, a high level of family drug knowledge and scientific literacy, so that the family pediatric drug tends to be rigorous and standardized [26]. The huge resources of the healthcare system support “peer-to-peer” personalized pediatric medical treatment plans, avoiding the problem of irrational or redundant pediatric medication. In addition, other scholars emphasize the issue of compounding and flexibility. That is to say, in pediatric medicine, we should consider children's patients in a forward-looking manner. Because children are in a complex external environment, the development of their own immune system is incomplete, and it is very likely to form cross or multiple infections. It is also difficult to keep track of the progress of the disease. This means that pediatric medications need to be considered in multiple ways in order to avoid potential medication conflicts and prevent future outbreaks.

According to the National Adverse Drug Reaction Monitoring Annual Report (2022), 7.8% of all adverse drug reaction reports in China are in children aged 14 years and younger. 86% of drug poisoning incidents in children occur during home medication use. Nearly 85% of families are at risk for children's medication safety. Focusing on children's pCms, the situation of few varieties, few dosage forms, few specifications and few labels needs to be reversed. Children's pCms are characterized by smaller dosage, relatively suitable taste and portability. However, there are few varieties of pCms for children, with only 12.6% of children's pCms included in the National Essential Drugs Catalog (2018 edition). Compared with the demand for children's medicines on the market, there are problems such as insufficient varieties in the supply of medicines. Relevant data show that the morbidity rate for children has been around 19.4% in recent years. However, only 3.2% of the drugs approved for marketing in China are specialized for children, and only 12.4% of the drugs are used by adults and children together. In cerebral, renal, dermatologic, and toxicological diseases, children-specific drugs are even more insufficient. China has established a more comprehensive urban and rural residents' medical insurance system, which is centered on the scientific construction of a pediatric medication system, as well as the maintenance and protection of the health and property interests of families. The number of insured children is steadily increasing, with 256 million insured children in 2023. The steady increase in the number of insured children has played a fundamental role in safeguarding the health rights and interests of children. At the same time, however, there are problems such as the many procedures and lengthy processes involved in insuring newborns, the lack of awareness among a small number of parents of the need to participate in the insurance system, and the fact that individual cities have not yet fully liberalized the restrictions on the household registration of resident children to participate in the insurance system. Although the government has invested more elements to serve the construction of pediatric medication system, the efficiency is still not high, and the quality of medication and the results of medication are not satisfactory [27]. It is particularly important to design a scientific pediatric medication evaluation structure system, and to measure and adjust the effectiveness of pediatric medication use in China based on this system.

Taken as a whole, time, price, efficacy, flexibility and safety are only some of the main focuses of pediatric drug use in developing countries. In reality, differences in subjects, environments and subjective and objective thinking have potentially shaped many of the guiding factors and key motivations for pediatric drug use. There is a need for an objective overview of the underlying causes of pediatric medication use based on the assessment process and attention to the integration of core operational and child and family needs. On the other hand, previous pediatric medication management lacked an application-friendly and process-oriented, cause-and-effect approach [28]. A detailed analysis of the current state of affairs is needed before assessment to show the complex process of pediatric medication administration. Based on this, this paper proposes to construct a pediatric medication diagnostic assessment framework with developing country characteristics and in line with the above regional conditions. In order to show the logic of hospitals, doctors, parents, and children in the context of resource constraints to use and receive medication. This is essentially a model of pediatric medication use based on lean management principles [29]. Through a well-conceived pediatric medication process model centered on optimizing resources and organizing and combing, it can improve the efficiency of pediatric medication administration in developing countries and alleviate the contradictions in the development of pediatrics. We refer to the lean management principles proposed to integrate the customer's desire (pediatric medication requirements in developing countries) with the process as a whole (pediatric medication-oriented cause and effect) to determine the priority strength of key factors in pediatric medication [30]. This provides an effect-oriented classification of pediatric medication root causes. Among them, MDM is a research method with good adaptability. Since pediatric medication use involves a variety of indicators such as processes and factors, the existence of MDM enables the joint judgment of multi-domain matrices and ultimately the assessment results.

Based on the assessment results, a scientific efficacy analysis model is needed to examine and verify the validity of the assessment and to analyze the pediatric medications in a profound and quantitative way based on the practice data. For a long time, DEA has been an effective method to measure the effectiveness results. DEA allows the axiomatic assumptions to be circumvented in assessment studies through the design of multi–input–multi–output systems. DEA floats segmented linear surfaces to the top of the data through linear programming techniques. In other words, statistical regression methods estimate parameters in the form of hypothetical functions by performing a single optimization over all decision-making units (DMUs). DEA, on the other hand, uses different optimizations (linear programming problems) for different DMUs and makes no a priori assumptions about the underlying functional form. Traditional DEA is dominated by radial characterization, i.e., it considers the relationship between inputs and outputs varying in the same proportion. This relationship determines the non-necessity of a priori information about the underlying function. In green governance activities, it is obvious that there is an ambiguity relationship between inputs and outputs. Traditional DEA efficiency is characterized by radial efficiency scores and possible non-zero input (output) slack that are difficult to retain. The radial Malmquist productivity index is based on radial DEA efficiency only. Ignoring non-zero input slack in an input-oriented index (or non-zero output slack in an output-oriented index) clearly does not fully characterize productivity changes [31]. The non-radial Malmquist index, on the other hand, effectively analyzes the efficacy results of complex scaling relationships. Considering the complexity of the MDM matrix assessment results and the unstructured nature of the real transformation assessment system, the non-radial DEA-Malmquist method effectively solves this problem. This enables accurate assessment of pediatric medication efficacy.

## Methods

### Research methods

**Fig. 1:**
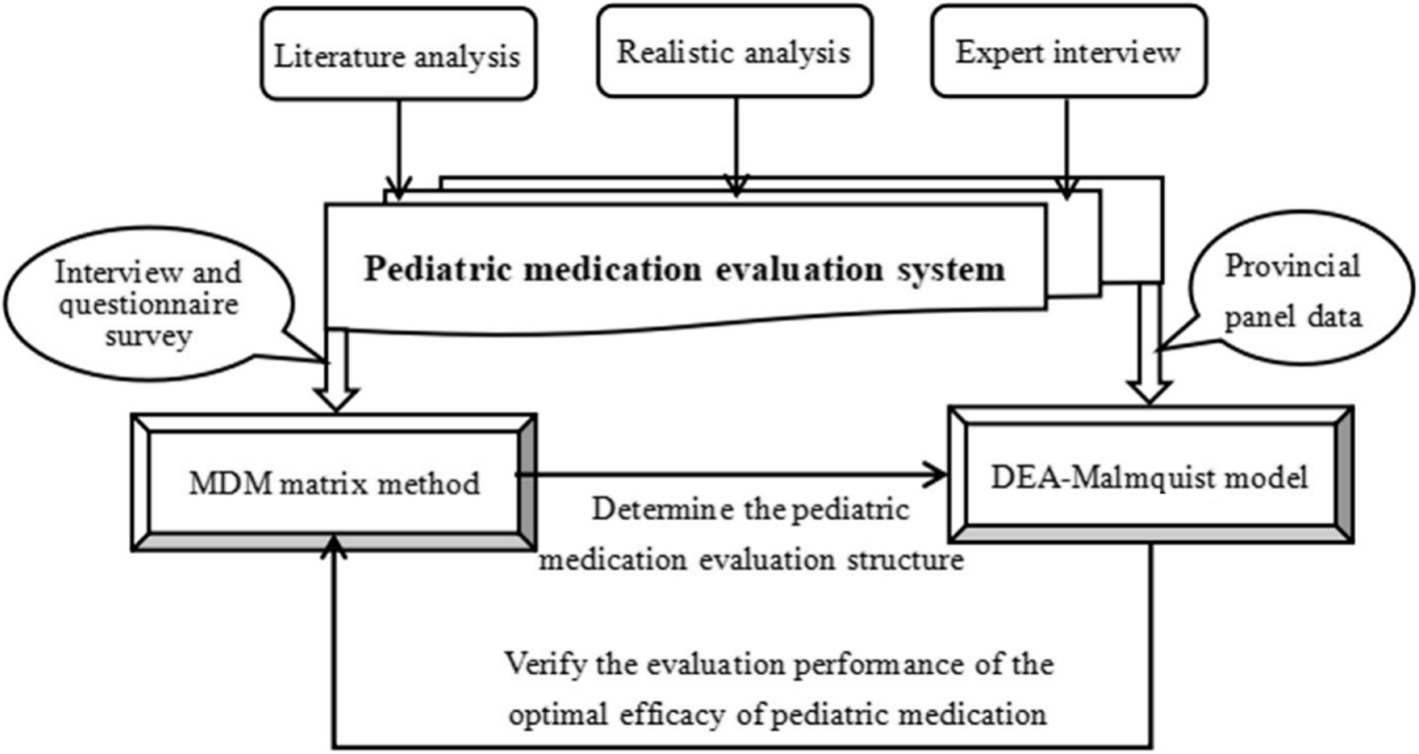
Pediatric medication evaluation modeling and analysis framework

This paper constructs and uses a joint model of MDM and DEA Malmquist to effectively analyze the current status and scientific improvement direction of pediatric medication in China. Its operational logic is shown in Fig. 1.

As shown in Fig. 1, based on pediatric medication literature and reality, combined with expert interviews, a multi-level and multi cycle evaluation index system for pediatric medication has been formed [32]. Based on small sample measurement and analysis, improve the MDM matrix framework and conduct actual calculations. After clarifying the multidimensional ranking of medication factors under each goal orientation, the indicators of each factor are mapped into an input–output system and input into the DEA Malmquist model, combined with panel data from each province for measurement. Based on the results of the DEA Malmquist model, verify whether the optimal performance of pediatric efficacy evaluation is consistent with the factor ranking of the MDM matrix framework [33], and also extend the effectiveness and practical strategies of pediatric medication evaluation results.

1. MDM Matrix Method

This study constructed a framework for assessing pediatric medication use in developing countries, drawing on the Lean Management Model and the MDM Matrix principles [34]. The structure of the framework is shown in Table 1. In this framework, PD (Pediatric direction) refers to the outcome factors, i.e., outcome variables, surrounding pediatric medication; MF (Main Factor) refers to the primary factors surrounding pediatric medicines, i.e., time, price, effectiveness, flexibility, and safety as analyzed in the previous section, and TF (Time Factor) refers to the time factors surrounding pediatric medication, including three factors: before, during, and after the drug is administered [35]. AF (Actual Factor) refers to the actual factors surrounding pediatric medicines. A multilevel causal structure within the Actual Factor is equally mapped and divided into different time periods (TFs). MAF*PD is characterized as the feedback of the Actual Factor on the final pediatric medication and is presented as a ranked order of the percentage of each factor, which exhibits the detailed importance of each factor for the reference of the physicians and the families. MMF*PD、MTF*MF、MAF*TF、MAF*AF are the scores of each level of the factor in the evaluation based on the expert research, where MTF*MF are computed using a seven-point Likert scale, as a way to differentiate the differences in the importance of each level of factors in each category [36].

**Table 1:** Framework for assessing pediatric medication use in developing countries

	PD	MF	TF		AF	
PD						
MF	MMF*PD					
TF		MTF*MF				
AF	MAF*PD	MAF*MF	MAF*TF	M*AF*TF	MAF*AF	M*AF*AF

Based on the MDM matrix framework, this paper can calculate the M_AF*PD_、M_AF*MF_、M*_AF*TF_、M*_AF*AF_ results, which are calculated as follows:

$$\begin{aligned} & M_{AF*AF}^{*} = M_{AF*TF} + M_{AF*AF}^{1} +\ldots M_{AF*TF}^{\text{n}} \\ & M_{AF*TF}^{*} = M_{AF*TF} + M_{AF*AF}^{*} \cdot M_{AF*TF} \\ & M_{AF*MF} = M_{AF*TF}^{*} \cdot M_{TF*MF}\\ & M_{AF*PD} = M_{AF*MF} \cdot M_{MF*PD} \end{aligned}$$

(2) DEA-malmquist model

For a long time, DEA (Data Envelopment Analysis) has been the main method for objective evaluation. DEA avoids axiomatic assumptions in evaluation research through the design of a multi input multi output system. In the evaluation of pediatric medication, its indicators cover many dimensions before, during, and after medication, but the induction of these dimensions should follow the principles of scientific logic. The combination of MDM framework and DEA model is beneficial for comprehensive coverage of indicators and optimization of internal evaluation structure, resulting in more scientific evaluation results. In the MDM framework, through screening principles, some variables are used as inputs to the DEA model, and the state and impact dimensions are used as outputs of the DEA model. Through the matching adjustment of this structure, the most authentic pediatric medication efficacy is clarified [37].

Considering that non-zero input slack in the input oriented index (or non-zero output slack in the output oriented index) cannot fully describe changes in pediatric medication. Therefore, this study extends the radial Malmquist index to a non radial index [38], where the input oriented index does not allow non-zero input relaxation and the output oriented index does not allow non-zero output relaxation. Assuming there are n decision units (DMUs) in the study, each DMUj generates a corresponding output $y_{j}^{t} = (y_{1j}^{t} ,...{,}y_{sj}^{t} )$ at each set of time t through a set of input $x_{j}^{t} = (x_{1j}^{t} ,...{,}x_{mj}^{t} )$ variables. Therefore, the most primitive DEA model is:

1 $$\begin{gathered} \theta_{0}^{t} (x_{0}^{t} ,y_{0}^{t} ) = \min_{{\theta_{0} ,\lambda_{j} }} \theta_{0} \hfill \\ s.t.{\kern 1pt} {\kern 1pt} {\kern 1pt} {\kern 1pt} {\kern 1pt} {\kern 1pt} \sum\limits_{j = 1}^{n} {\lambda_{j} x_{j}^{t} } \le \theta_{0} x_{0}^{t} , \hfill \\ {\kern 1pt} {\kern 1pt} {\kern 1pt} {\kern 1pt} {\kern 1pt} {\kern 1pt} {\kern 1pt} {\kern 1pt} {\kern 1pt} {\kern 1pt} {\kern 1pt} {\kern 1pt} {\kern 1pt} {\kern 1pt} {\kern 1pt} {\kern 1pt} {\kern 1pt} \sum\limits_{j = 1}^{n} {\lambda_{j} y_{j}^{t} } \ge y_{0}^{t} \hfill \\ {\kern 1pt} {\kern 1pt} {\kern 1pt} {\kern 1pt} {\kern 1pt} {\kern 1pt} {\kern 1pt} {\kern 1pt} {\kern 1pt} {\kern 1pt} {\kern 1pt} {\kern 1pt} {\kern 1pt} {\kern 1pt} {\kern 1pt} {\kern 1pt} \lambda_{j} \ge 0,j = 1,...,n \hfill \\ \end{gathered}$$

Model (1) is an input oriented formula that considers the potential radial reduction trend of output factors at the current level of output scale. By replacing $x_{j}^{t + 1}$ $y_{j}^{t + 1}$ and $x_{j}^{t}$ $y_{j}^{t}$ studying, the technical efficiency of each decision-making unit can be obtained during the t + 1 period. Through the update from period t to period t + 1, the technical efficiency of each decision-making unit will change, and the forefront of experience production will also change accordingly. Research has formed the following non radial DEA [39]:

2 $$\begin{gathered} \tilde{\theta }_{0}^{t} (x_{0}^{t} ,y_{0}^{t} ) = \frac{1}{{\sum\limits_{i = 1}^{m} {\alpha_{i} } }}\min_{{\theta_{{_{0} }}^{i} ,\lambda_{j} }} \sum\limits_{i = 1}^{m} {\alpha_{i} } \theta_{0}^{i} \hfill \\ s.t.{\kern 1pt} {\kern 1pt} {\kern 1pt} {\kern 1pt} {\kern 1pt} {\kern 1pt} \sum\limits_{j = 1}^{n} {\lambda_{j} x_{ij}^{t} } \le \theta_{0}^{i} x_{i0}^{t} ,{\kern 1pt} {\kern 1pt} {\kern 1pt} {\kern 1pt} i = 1,...,m \hfill \\ {\kern 1pt} {\kern 1pt} {\kern 1pt} {\kern 1pt} {\kern 1pt} {\kern 1pt} {\kern 1pt} {\kern 1pt} {\kern 1pt} {\kern 1pt} {\kern 1pt} {\kern 1pt} {\kern 1pt} {\kern 1pt} {\kern 1pt} {\kern 1pt} {\kern 1pt} \sum\limits_{j = 1}^{n} {\lambda_{j} y_{rj}^{t} } \ge y_{r0}^{t} ,{\kern 1pt} {\kern 1pt} {\kern 1pt} {\kern 1pt} {\kern 1pt} r = 1,...s, \hfill \\ {\kern 1pt} {\kern 1pt} {\kern 1pt} {\kern 1pt} {\kern 1pt} {\kern 1pt} {\kern 1pt} {\kern 1pt} {\kern 1pt} {\kern 1pt} {\kern 1pt} {\kern 1pt} {\kern 1pt} {\kern 1pt} {\kern 1pt} {\kern 1pt} {\kern 1pt} \theta_{i}^{0} {\kern 1pt} {\kern 1pt} free \hfill \\ {\kern 1pt} {\kern 1pt} {\kern 1pt} {\kern 1pt} {\kern 1pt} {\kern 1pt} {\kern 1pt} {\kern 1pt} {\kern 1pt} {\kern 1pt} {\kern 1pt} {\kern 1pt} {\kern 1pt} {\kern 1pt} {\kern 1pt} {\kern 1pt} \lambda_{j} \ge 0,j = 1,...,n. \hfill \\ \end{gathered}$$

The specified weights are $a_{i}$ i = 1,…, m, to reflect the different preferences of each decision-making unit for improving various inputs. Model (2) measured the relative efficiency of DMU during time period t under weight $a_{i}$ guidance. If $a_{i}$ = 0 and the corresponding $\theta_{i}^{0}$ = 1 is set. At this point, the greater the weight, the higher the priority given by the DMU to reduce its i-th input. Thus, model (2) determined the optimal EPF. Corresponding to the characterization of Malmquist productivity index in radial DEA, the relative efficiency of DMU at time t + 1 can be obtained:

3 $$\begin{gathered} \tilde{\theta }_{0}^{t + 1} (x_{0}^{t} ,y_{0}^{t} ) = \frac{1}{{\sum\limits_{i = 1}^{m} {\alpha_{i} } }}\min_{{\theta_{{_{0} }}^{i} ,\lambda_{j} }} \sum\limits_{i = 1}^{m} {\alpha_{i} } \theta_{0}^{i} \hfill \\ s.t.{\kern 1pt} {\kern 1pt} {\kern 1pt} {\kern 1pt} {\kern 1pt} {\kern 1pt} \sum\limits_{j = 1}^{n} {\lambda_{j} x_{ij}^{t + 1} } \le \theta_{0}^{i} x_{i0}^{t} ,{\kern 1pt} {\kern 1pt} {\kern 1pt} {\kern 1pt} i = 1,...,m \hfill \\ {\kern 1pt} {\kern 1pt} {\kern 1pt} {\kern 1pt} {\kern 1pt} {\kern 1pt} {\kern 1pt} {\kern 1pt} {\kern 1pt} {\kern 1pt} {\kern 1pt} {\kern 1pt} {\kern 1pt} {\kern 1pt} {\kern 1pt} {\kern 1pt} {\kern 1pt} \sum\limits_{j = 1}^{n} {\lambda_{j} y_{rj}^{t + 1} } \ge y_{r0}^{t} ,{\kern 1pt} {\kern 1pt} {\kern 1pt} {\kern 1pt} {\kern 1pt} r = 1,...s, \hfill \\ {\kern 1pt} {\kern 1pt} {\kern 1pt} {\kern 1pt} {\kern 1pt} {\kern 1pt} {\kern 1pt} {\kern 1pt} {\kern 1pt} {\kern 1pt} {\kern 1pt} {\kern 1pt} {\kern 1pt} {\kern 1pt} {\kern 1pt} {\kern 1pt} {\kern 1pt} \theta_{i}^{0} {\kern 1pt} {\kern 1pt} free \hfill \\ {\kern 1pt} {\kern 1pt} {\kern 1pt} {\kern 1pt} {\kern 1pt} {\kern 1pt} {\kern 1pt} {\kern 1pt} {\kern 1pt} {\kern 1pt} {\kern 1pt} {\kern 1pt} {\kern 1pt} {\kern 1pt} {\kern 1pt} {\kern 1pt} \lambda_{j} \ge 0,j = 1,...,n. \hfill \\ \end{gathered}$$

Furthermore, by replacing the t + 1 values of input and output variables, the relative efficiency and EPF at time t are obtained

4 $$\begin{gathered} \tilde{\theta }_{0}^{t} (x_{0}^{t + 1} ,y_{0}^{t + 1} ) = \frac{1}{{\sum\limits_{i = 1}^{m} {\alpha_{i} } }}\min_{{\theta_{{_{0} }}^{i} ,\lambda_{j} }} \sum\limits_{i = 1}^{m} {\alpha_{i} } \theta_{0}^{i} \hfill \\ s.t.{\kern 1pt} {\kern 1pt} {\kern 1pt} {\kern 1pt} {\kern 1pt} {\kern 1pt} \sum\limits_{j = 1}^{n} {\lambda_{j} x_{ij}^{t} } \le \theta_{0}^{i} x_{i0}^{t + 1} ,{\kern 1pt} {\kern 1pt} {\kern 1pt} {\kern 1pt} i = 1,...,m \hfill \\ {\kern 1pt} {\kern 1pt} {\kern 1pt} {\kern 1pt} {\kern 1pt} {\kern 1pt} {\kern 1pt} {\kern 1pt} {\kern 1pt} {\kern 1pt} {\kern 1pt} {\kern 1pt} {\kern 1pt} {\kern 1pt} {\kern 1pt} {\kern 1pt} {\kern 1pt} \sum\limits_{j = 1}^{n} {\lambda_{j} y_{rj}^{t} } \ge y_{r0}^{t + 1} ,{\kern 1pt} {\kern 1pt} {\kern 1pt} {\kern 1pt} {\kern 1pt} r = 1,...s, \hfill \\ {\kern 1pt} {\kern 1pt} {\kern 1pt} {\kern 1pt} {\kern 1pt} {\kern 1pt} {\kern 1pt} {\kern 1pt} {\kern 1pt} {\kern 1pt} {\kern 1pt} {\kern 1pt} {\kern 1pt} {\kern 1pt} {\kern 1pt} {\kern 1pt} {\kern 1pt} \theta_{i}^{0} {\kern 1pt} {\kern 1pt} free \hfill \\ {\kern 1pt} {\kern 1pt} {\kern 1pt} {\kern 1pt} {\kern 1pt} {\kern 1pt} {\kern 1pt} {\kern 1pt} {\kern 1pt} {\kern 1pt} {\kern 1pt} {\kern 1pt} {\kern 1pt} {\kern 1pt} {\kern 1pt} {\kern 1pt} \lambda_{j} \ge 0,j = 1,...,n. \hfill \\ \end{gathered}$$

Finally, through $\theta_{i}^{0}$ ($x_{0}^{t}$, $y_{0}^{t}$) 、$\theta_{0}^{t + 1}$ ($x_{0}^{{t + {1}}}$, $y_{0}^{{t + {1}}}$) 、$\theta_{0}^{t + 1}$ ($x_{0}^{t}$, $y_{0}^{t}$) 和 $\theta_{i}^{0}$ ($x_{0}^{{t + {1}}}$, $y_{0}^{{t + {1}}}$) The non radial efficiency score determines the input oriented non radial Malmquist productivity index:

$$P\tilde{I}_{0} = \frac{{\tilde{\theta }_{0}^{t} (x_{0}^{t} ,y_{0}^{t} )}}{{\tilde{\theta }_{0}^{t + 1} (x_{0}^{t + 1} ,y_{0}^{t + 1} )}}\left[ {\frac{{\tilde{\theta }_{0}^{t + 1} (x_{0}^{t + 1} ,y_{0}^{t + 1} )\tilde{\theta }_{0}^{t + 1} (x_{0}^{t} ,y_{0}^{t} )}}{{\tilde{\theta }_{0}^{t} (x_{0}^{t + 1} ,y_{0}^{t + 1} )\tilde{\theta }_{0}^{t} (x_{0}^{t} ,y_{0}^{t} )}}} \right]^{1/2}$$

### Data acquisition

**Fig. 2:**
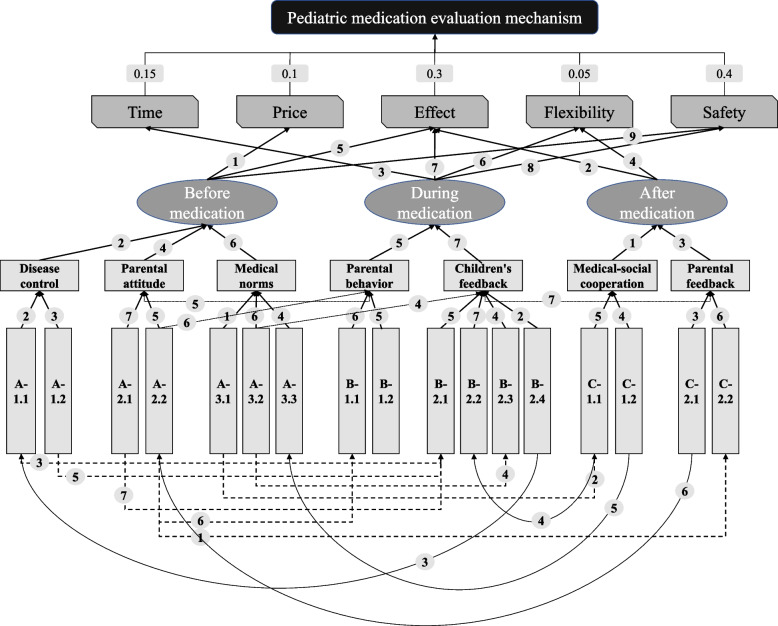
The relationship between the indicators of pediatric medication evaluation

In this paper, based on pediatric general hospitals and healthcare disciplines, health system experts' research, and a literature review, 5 MFs, 3 TFs, 7 level 1 AFs and 17 level 2 AFs frameworks were formed. Based on the research of 20 pediatric experts and 30 groups of families, the strong and weak relationships were obtained after mean transformation and ordinal comparison, as shown in Fig. 2. Among them, the arrows indicate the transmission of causality, and the symbols on the arrows show the relative strength of the causal chain, e.g., "7" shows more robust causality than "5", meaning that pediatric medication should pay more attention to this factor [40–42].

The meanings of the symbols in Fig. 2 are as follows [43–45].

- A-1.1:Common diseases; A-1.2:Disease severity; A-2.1:Childhood status; A-2.2:Parental cognition;
- A-3.1:Hospital infrastructure; A-3.2:Doctor's Literacy; A-3.3:Doctor preferences.
- B-1.1:Timeliness of medication; B-1.2:Drug control degree;
- B-2.1:Improvement rate of children;B-2.2:Child Medication;B-2.3:Disease variability;B-2.4:external environment.
- C-1.1:Health records;C-1.2:Medication tracking;C-2.1:Health habits;C-2.2:Medical network.

Furthermore, based on the analysis logic and core variables in Fig. 2, combined with causal relationships and input–output perspectives, the data is realistically extracted and summarized into the input–output framework of the DEA model. The variables are shown in Table 2. More details are shown in Appendix A.

**Table 2:** Variables for analysis of pediatric medication efficacy in Chinese Provinces

Evaluation dimension	Evaluation index	Representational index	Data source
Input	External environment	Expenditure on medical and health care Number of tertiary pediatric hospitals Pediatric medicine and health website	(1) (2) (2)
	Hospital infrastructure	Number of beds in pediatric hospitals	(1)
	Doctor's Literacy	Number of pediatricians with senior titles	(2)
	Doctor preferences	Number of pediatric pharmacists with professional qualifications	(2)
	Drug control degree	Health training of pediatric medicine	(2)
	Medication tracking	Pediatrician patient load ratio	(2)
	Health habits	Pediatric medicine technical consultation	(2)
	Health records	Public health records management	(2)
Output	Improvement rate of children	Number of pediatric discharges	(1)
	Disease variability	Number of pediatric outpatient and emergency patients	(1)
	Medication tracking	The scale of pediatric use	(1)
	Common diseases	Incidence of pediatric category A and B diseases	(2)
	Disease severity	Mortality from pediatric category A and B diseases	(2)
	Childhood status	Scale of pediatric family health services	(2)
	Parental cognition	Effective child support ratio	(2)
	Medical network	Community home medication guidance	(2)

(1) China Statistical Yearbook, (2) China Health Statistics Yearbook

## Results

### MDM matrix analysis results

#### Key capture of overall medication factors

Based on the results of the data survey (Fig. 2), in developing countries, pediatricians and parents of affected children generally focus on drug prices, efficacy, and safety during the pre medication period [46]. Due to differences in symptom characteristics and limitations in medical conditions, pediatric medication in developing countries generally does not overly emphasize timing and compatibility issues before use. As the medication phase begins, families of affected children begin to pay attention to medication timing, efficacy, flexibility, and safety issues. In terms of medication duration, families are generally concerned that prolonged medication cycles may harm children's bodies or have adverse effects. In terms of medication flexibility, children generally have more changes in their condition, such as fluctuating fever or common diarrhea problems, which often worsen or are mixed in with the original condition. And parents cannot go to the hospital all the time to confirm the medication status and coordinate with doctors to modify the prescription, which leads to parents temporarily increasing medication. The mixing of multiple drugs highlights the issue of medication flexibility in children, and parents must pay attention to the conflicts and compatibility mechanisms of medication when applying it. In the post medication stage, medical institutions and families generally continue to pay attention to the effectiveness and flexibility of medication [47]. On the one hand, the effectiveness of medication lies in the manifestation of medication integrity. Families of sick children are generally concerned about the possibility of recurrent illness and medication failure, which may further damage the child's health. On the other hand, some children with chronic diseases also need to worry about the flexibility of medication left after use [48]. Based on research and questionnaire collection evaluation results, the importance ratios of time, price, effectiveness, flexibility, and safety are 0.15, 0.1, 0.3, 0.05, and 0.4. During the specific period, safety assessment before medication is the most important, followed by safety considerations during the medication phase. The importance of pediatric medication efficacy is slightly weaker during the medication phase, followed by consideration of flexibility during medication, analysis of efficacy before medication, and consideration of flexibility after medication [49].

#### Capture the key factors of actual medication use

In the actual factor representation, the pre-medication phase was divided into three primary sub-factor constructs: disease control, parental attitudes, and physician norms; the mid-medication phase included two sub-factor constructs: parental behavior and child feedback; and the post-medication cycle was divided into two sub-factor constructs: medical-social cooperation and parental feedback [50].

In the premedication cycle. Disease control is primarily a normative code for the use of medication in children. Among other things, the commonness of the disease and the severity of the child's illness affect the effectiveness of the manual and ultimately determine the normative nature of disease control before medication administration. Parental attitudes are also a critical factor in the premedication assessment, which consists of two sub-dimensions: child status and parental perceptions. The reality of the child's state before the medication visit can profoundly affect the final medication pattern. If a child's state is poor, such as depression and loss of appetite, even if their condition is not severe and the disease is not complex, it will affect the doctor's prescription of medication [51]. In addition, parental perception is an essential factor in medication assessment. Low parental literacy or poor medication habits may start doctor-patient conflicts. In addition, misperceptions may force physicians to forgo the use of fast and efficient pediatric medications. Physician norms are also a key consideration in the premedication cycle. Hospital infrastructure, physician literacy, and physician preference will ultimately influence the expression of physician norms. In China, high-level hospitals such as tertiary hospitals are in better condition to support the cultivation of physician's medication specifications.

Medication-in-use phase. Parental behavior and child feedback determine the state of medication assessment. Due to the unique nature of pediatric medication administration, parental behavior is critical in guiding medication administration and exerting efficacy. Parental behavior includes medication punctuality and dosage control. Therefore, parental habits and busyness should be considered in pediatric medication assessment to adjust medication protocols and avoid multiple doses, multiple dosages, or complex means of medication administration. In addition, children's feedback is an important category to consider. Children getting better, children being medicated, and changes in disease all determine the outcome of children's feedback and determine whether parents or physicians need to adjust pediatric medications. In addition, the external environment can accelerate thinking about pediatric medicines. For example, in the context of the new crown, children may exacerbate disease adjustments, thus requiring multidimensional consideration of medication.

In the post-medication cycle, developing countries need help to build solid medical community systems need be built. Therefore, the emerging health records and the tracking of national medication habits determine the medical community's cooperation quality and ultimately affect the pediatric medication mechanism. In China, in recent years, health administrations have invested much effort in tracking and managing common pediatric medications to circumvent the abuse of some inefficient or inappropriate medications. With the continued promotion of medical community building, pediatric medication assessment has become more standardized and proactive, and the quality of medication management has improved dramatically. In addition, parental feedback is a vital assessment variable, and their health care habits and medical contacts, developed over time, can profoundly determine the outcome of a family's pediatric medication use [52].

In addition, some of the second-level practical factors also influence each other and even feedback or indirectly influence other first-level practical factors. The specific assessment results are shown in Fig. 2. Based on MDM structure establishment and data analysis calculation, this paper has formed the core factor results of pediatric medication evaluation, as shown in Table 3.

**Table 3:** Results of the relationship between pediatric medication use assessments in developing countries

		PD	MF					TF			AF							
			Time	Price	Effect	Flexibility	Safety	A	B	C	A-1	A-1.1	A-1.2	A-2	A-2.1	A-2.2	A-3	A-3.1
PD																		
MF	Time	0.15																
	Price	0.1																
	Effect	0.3																
	Flexibility	0.05																
	Safety	0.4																
TF	Before A			1	5		9											
	During B		3		7	6	8											
	After C				2	4												
AF	A-1	-----	0	0	0.169	0	0	0	0	0	0	0	0	0	0	0	0	0
	A-1.1	0.031	0.070	0.343	0.262	0.067	0.176	0.244	0.070	0.023	0.8	0	0	0	0	0	0	0
	A-1.2	0.045	0.116	0.514	0.335	0.111	0.270	0.367	0.116	0.038	1.2	0	0	0	0	0	0	0
	A-2	-----	0.498	0	0.560	0.391	0.254	0	0.498	0	0	0	0	0	0	0	0	0
	A-2.1	0.108	0.308	1	1	0.270	0.566	0.713	0.308	0.053	0	0	0	1.167	0	0	0	0
	A-2.2	0.094	1	0.714	0	1.010	0.802	0.509	1	0.429	0	0	0	0.833	0	0	0	0
	A-3	-----	0	0	0.480	0	0	0	0	0	0	0	0	0	0	0	0	0
	A-3.1	0.093	0.488	0.234	1.078	0.578	0.344	0.167	0.488	0.371	0	0	0	0	0	0	0.182	0
	A-3.2	0.126	0.837	1.403	0.320	0.753	1	1	0.837	0.182	0	0	0	0	0	0	1.091	0
	A-3.3	0.039	0	0.935	0	0	0.382	0.667	0	0	0	0	0	0	0	0	0.727	0
	B-1	-----	0	0	0.402	0	0	0	0	0	0	0	0	0	0	0	0	0
	B-1.1	0.053	0.598	0	0.335	0.469	0.305	0	0.598	0	0	0	0	0	0	0	0	0
	B-1.2	0.040	0.498	0	0.192	0.391	0.254	0	0.498	0	0	0	0	0	0	0	0	0
	B-2	-----	0	0	0.491	0.524	0	0	0	1	0	0	0	0	0	0	0	0
	B-2.1	0.076	0.697	0	0.687	0.607	0.355	0	0.697	0.114	0	0	0	0	0	0	0	0
	B-2.2	0.080	0.976	0	0.393	0.850	0.498	0	0.976	0.159	0	0	0	0	0	0	0	0
	B-2.3	0.049	0.558	0	0.306	0.486	0.284	0	0.558	0.091	0	0	0	0	0	0	0	0
	B-2.4	0.029	0.349	0.171	0	0.310	0.248	0.122	0.349	0.068	0.4	2	0	0	0	0	0	0
	C-1	-----	0	0	0.440	0	0	0	0	0	0	0	0	0	0	0	0	0
	C-1.1	0.043	0.488	0	0.225	0.688	0.249	0	0.488	0.582	0	0	0	0	0	0	0	0
	C-1.2	0.021	0	0.468	0	0.177	0.191	0.333	0	0.339	0	0	0	0	0	0	0.364	0
	C-2	-----	0	0	0.759	0	0	0	0	0	0	0	0	0	0	0	0	0
	C-2.1	0.070	0.701	0.357	0.165	1	0.504	0.255	0.701	0.857	0	0	0	0.417	0	2	0	0
	C-2.2	0.004	0	0	0	0.449	0	0	0	0.857	0	0	0	0	0	0	0	0

		AF															
		A-3.2	A-3.3	B-1	B-1.1	B-1.2	B-2	B-2.1	B-2.2	B-2.3	B-2.4	C-1	C-1.1	C-1.2	C-2	C-2.1	C-2.2
PD																	
MF	Time																
	Price																
	Effect																
	Flexibility																
	Safety																
TF	Before A																
	During B																
	After C																
AF	A-1	0	0	0	0	0	0	0	0	0	0	0	0	0	0	0	0
	A-1.1	0	0	0	0	0	0.045	0.4	0	0	0	0	0	0	0.020	0	0
	A-1.2	0	0	0	0	0	0.076	0.667	0	0	0	0	0	0	0.033	0	0
	A-2	0	0	0.455	0	0	0	0	0	0	0	0	0	0	0	0	0
	A-2.1	0	0	0.133	0	0	0.106	0.933	0	0	0	0	0	0	0.046	0	0
	A-2.2	0	0	0.913	2	0	0	0	0	0	0	0	0	0	0.375	0	2
	A-3	0	0	0	0	0	0	0	0	0	0	0	0	0	0	0	0
	A-3.1	0	0	0	0	0	0.318	0	1	0	0	0.556	2	0	0.139	0	0
	A-3.2	0	0	0	0	0	0.545	0	0	2	0	0	0	0	0.159	0	0
	A-3.3	0	0	0	0	0	0	0	0	0	0	0	0	0	0	0	0
	B-1	0	0	0	0	0	0	0	0	0	0	0	0	0	0	0	0
	B-1.1	0	0	0.545	0	0	0	0	0	0	0	0	0	0	0	0	0
	B-1.2	0	0	0.455	0	0	0	0	0	0	0	0	0	0	0	0	0
	B-2	0	0	0	0	0	0	0	0	0	0	0	0	0	0.875	0	0
	B-2.1	0	0	0	0	0	0.455	0	0	0	0	0	0	0	0.099	0	0
	B-2.2	0	0	0	0	0	0.636	0	0	0	0	0	0	0	0.139	0	0
	B-2.3	0	0	0	0	0	0.364	0	0	0	0	0	0	0	0.080	0	0
	B-2.4	0	0	0	0	0	0.227	0.2	0	0	0	0	0	0	0.060	0	0
	C-1	0	0	0	0	0	0	0	0	0	0	0	0	0	0	0	0
	C-1.1	0	0	0	0	0	0.318	0	2	0	0	1.111	0	0	0.139	0	0
	C-1.2	0	2	0	0	0	0	0	0	0	0	0.889	0	0	0	0	0
	C-2	0	0	0	0	0	0	0	0	0	0	0	0	0	0	0	0
	C-2.1	0	0	0.640	1	0	0	0	0	0	0	0	0	0	0.75	0	1
	C-2.2	0	0	0	0	0	0	0	0	0	0	0	0	0	0.75	0	0

The following results can be obtained from Table 3. Specifically, The proportion of factor assessment before medication is the highest, reaching 53.5%; Next is the pediatric evaluation during the mid-term of medication, with factor evaluation accounting for approximately 32.7%; The proportion of pediatric evaluation factors in the post medication cycle is about 13.7%. However, it is worth noting that the number of evaluation factors in the post medication stage of pediatrics is the smallest, only 4, so its average evaluation proportion is 3.4%. The evaluation of various factors during medication accounts for 5.5%, while the evaluation of various factors before medication accounts for approximately 7.6%. This indicates that the evaluation of pediatric drug use in the pre medication stage is the most important and requires the most comprehensive consideration, with each stage accounting for the highest proportion of the overall evaluation. From the perspective of first level practical factors, the proportion of standardized evaluations by doctors is the highest, reaching 25.8%. The average proportion of secondary factors is 8.6%. Indicating that physician standardization is the most critical dimension in pediatric medication evaluation. The standardization of doctors and the quality of medication determine the final effectiveness of medication. The evaluation proportion of children's feedback is second only to doctor's standards, with a proportion of 23.5% and an average proportion of 5.9% in its sub dimensions. Therefore, the actual performance in pediatric medication is also a key factor for inclusion in the pediatric medication evaluation system. But the importance of its sub items is not as significant as the doctor's standard dimension. The key to improving the quality of medication lies in the matching and effective interaction between doctors and patients. The evaluation of parental attitudes accounts for 20.17% of the overall consideration, but its sub item proportion reaches 10.1%, indicating that child status and parental cognition constitute important evaluation sub variables. The proportions of disease control, parental behavior, medical and social cooperation, and parental feedback are all less than 10%, accounting for 7.6%, 9.3%, 6.4%, and 7.3% respectively, indicating that the above-mentioned AF is an important reference variable that plays a corrective and maintenance role in pediatric medication evaluation. From the perspective of secondary AF, physician competence is the most important factor, accounting for 12.6% of the total evaluation factors, followed by child status, accounting for 10.8%. The proportion of factors related to parental cognition, hospital infrastructure, medication punctuality, child improvement, child drug resistance, and health habits all exceeded 5%, accounting for9.4%, 9.3%, 5.3%, 7.6%, 8.0%, and 7.0%, respectively. For hospitals and health management departments, this indicator system can be used to input actual data and evaluate the effectiveness, combined with the proportion of each factor to obtain the final score of pediatric medication, in order to optimize the quality of various punishments and medication habits.

#### Capturing medication factors under different guiding objectives

In practice, due to increased constraints in medication administration, hospitals and doctors can optimize and adjust according to the specific claims of families. For example, if some families pursue the duration of medication, they can be optimized according to the AF-MF matrix.

Specifically, parental perception was the most critical and primary reference variable when pursued with medication duration. This is also more consistent with reality. The lack of parental perception can lead to conflicts between the doctor and the family, and too much strife and medication violation (not exercised according to the prescription in the actual medication by the parents) can lead to a failure in the assessment of the medication. Child receptivity to drugs is the second most crucial factor, with about 97.6% of the importance of the reference variable (parental perception). Good medication acceptance in children accelerates the time to take medication and avoids prolonged medication procrastination or dependence. The remaining essential factors were physician literacy (83.7%), health records (70.1%), child status (69.7%), medication punctuality (59.8%), and disease variability (55.8%), with less than 50% of the remaining factors. This is highly consistent with the reality that more competent doctors tend to cure the disease and shorten the medication cycle; the establishment of a health record helps to share some of the data of the child, which can lead to a suitable prescription for future medication; the status of the child is an important starting point for medication, and milder illnesses contribute to a quicker recovery; and punctuality of medication and changes in disease are also closely related to the length of drugs.

Child status is the primary reference variable when pursuing the price of medication. The child's status determines whether or not low-cost medicines can be administered. If the status of the child is too poor, it isn't very sensible to pursue the price of medication. On the other hand, physician literacy is the most crucial variable, with an importance share relative to the reference variable of 1.4. The remaining essential factors are physician preference (93.5%), parental perceptions (71.4%), and disease severity (51.4%). Doctors' medication habits determine the choice for drug prices, in addition to parental perceptions and disease severity, which determine the eventual use of cheaper drugs.

When the effect of medication is pursued, the child's status remains the primary reference variable. It is clear that the purpose of medication use, in addition to addressing childhood illnesses, is also primarily aimed at alleviating the status of the child. Hospital infrastructure is the most crucial variable, with an importance weight of 1.1 compared to the reference variable, and it is clear that in developing countries, hospital infrastructure determines the final effect of medication. This aligns with reality, so most families visit tertiary or pediatric specialist hospitals. High-quality hospital infrastructure and a high reputation of hospital prestige will attract many patients to visit the hospital. In addition, a higher level of hospital infrastructure determines the top pediatric resources in the developing world, which can generally support most pediatric patients' medication claims. The remaining factors are relatively close in level but slightly less important.

The health profile is the primary reference variable when medication flexibility is sought. An active and effective health record is a critical factor in the relationship between contraindications and flexibility of medication use in children. On the one hand, timely recording of children's medication habits, medication characteristics, and common acute and chronic diseases will help doctors avoid adverse compounding problems in subsequent medication administration; at the same time, a solid health record will help doctors quickly perceive children's characteristics, thus strengthening the flexibility of medication administration. Parental perception is the most critical factor, with a weighting of 1.01 compared to the reference variable, which means that parents are still the first person responsible for children's medication. Parents are often familiar with their children's characteristics and common illnesses and are aware of their children's allergies or medications with high side effects. These will help to avoid risks in pediatric medicines.

Physician literacy is the primary reference variable when medication safety is pursued and the most important one. Doctors possess the most knowledge about pediatric medication and are the most critical factor in achieving pediatric medication safety. Parental cognition was second only to physician literacy, with 80.2% importance. Positive parental cognition helps them to cooperate with the doctor, thus improving medication safety. Better family literacy can circumvent the problems of unauthorized and incorrect use of medication by parents and further enhance medication safety. The percentage of importance of hospital infrastructure (56.6%) and healthcare habits (50.4%) is higher than 50%, and the above factors positively affect medication safety outcomes.

Overall, despite the constraints on pediatric drug use and the underdeveloped level of care in developing countries, the goals of effective pediatric drug use can be achieved mainly with the help of the present assessment framework. However, with the help of this assessment framework, the goal of effective pediatric medication use can still be primarily achieved. By identifying multidimensional factor criticality, doctors and parents can quickly identify the key factors, accelerate the turnaround efficiency of pediatric consultation, improve the quality of pediatric medication, and promote the optimal construction of pediatrics in developing countries.

### Analysis of DEA Malmquist model

Furthermore, the evaluation variables will be macroscopically evaluated and combined with DEA model analysis to obtain the Malmquist index of pediatric medication in various provinces of China. As shown in Table 4.

**Table 4:** Malmquist index for pediatric medication in China

DMU	2015 = > 2016	2016 = > 2017	2017 = > 2018	2018 = > 2019	2019 = > 2020	2020 = > 2021	Average
Beijing	1.3474	0.9401	1.1579	0.8678	0.9907	1.3861	1.1150
Tianjin	1.0608	1.0507	0.8043	1.0471	0.9977	0.8367	0.9662
Hebei	1.0321	0.9063	1.0110	1.1700	0.8415	0.9620	0.9871
Shanxi	1.1121	2.2595	1.3814	0.8369	0.9151	0.6582	1.1939
Neimenggu	1.0831	1.0082	0.7754	1.0301	1.1141	1.0079	1.0031
Liaoning	1.0371	1.0654	0.9468	1.0338	0.8026	1.0248	0.9851
Jilin	1.1143	1.1543	0.8902	1.2602	1.1391	0.9545	1.0854
Heilongjiang	1.0099	0.9891	0.9792	0.9991	1.1967	0.7822	0.9927
Shanghai	1.0582	0.8512	1.0572	0.9407	0.8056	1.2851	0.9996
Jiangsu	0.9037	1.3239	1.7101	1.0898	0.9027	0.6927	1.1038
Zhejiang	0.9227	1.0934	1.0194	0.9934	0.6624	1.6944	1.0643
Anhui	0.9481	1.2625	0.8385	1.6745	0.8909	0.9083	1.0871
Fujian	1.0354	1.1314	0.8605	1.1568	0.8192	1.4026	1.0677
Jiangxi	1.0051	1.0312	0.9775	0.9628	0.9124	1.0618	0.9918
Shandong	0.9659	1.0209	1.0860	1.0275	0.9217	1.3509	1.0621
Henan	1.0604	1.0732	1.1041	0.8972	0.8572	1.0912	1.0139
Hubei	0.4151	1.0553	1.0067	0.9832	0.6863	1.2929	0.9066
Hunan	1.0722	1.0741	1.0364	1.0052	0.8853	1.0472	1.0201
Guangdong	0.9668	1.3910	0.8990	1.2463	0.7075	1.0987	1.0515
Guangxi	1.0026	1.2056	0.8290	1.0753	0.8517	1.0469	1.0019
Hainan	0.9085	1.0100	0.8613	0.9254	0.8388	1.1529	0.9495
Chongqing	0.9355	1.0840	0.9596	1.1555	0.8082	1.0860	1.0048
Sichuan	1.0046	1.1778	1.2952	1.1521	0.8900	1.3233	1.1405
Guizhou	0.8683	1.5661	1.0584	0.9208	1.0030	0.8453	1.0436
Yunnan	1.2020	0.9265	0.9845	1.2296	0.7724	0.9028	1.0030
Xizang	0.8638	0.8573	1.6086	0.6851	1.0643	0.8955	0.9958
Shaanxi	0.9887	1.0285	1.0348	0.9607	0.7798	1.1197	0.9854
Gansu	0.9903	0.9881	1.0121	1.0105	0.9126	1.0341	0.9913
Qinghai	1.0047	1.0697	0.9212	0.9309	0.9645	0.9793	0.9784
Ningxia	0.8560	0.9730	0.8111	1.1555	0.9584	0.8313	0.9309
Xinjiang	0.7389	1.7106	0.7675	1.0576	0.7900	1.0457	1.0184
Average	0.9843	1.1380	1.0221	1.0478	0.8930	1.0581	1.0239
Max	1.3474	2.2595	1.7101	1.6745	1.1967	1.6944	1.1939
Min	0.4151	0.8512	0.7675	0.6851	0.6624	0.6582	0.9066
SD	0.1534	0.2809	0.2200	0.1722	0.1266	0.2258	0.0619

As shown in Table 4, the average annual Malmquist index in Beijing, Shanxi, Inner Mongolia, Jilin, Jiangsu, Zhejiang, Anhui, Anhui, Anhui, Shandong, Henan, Hunan, Guangdong, Guangxi, Chongqing, Sichuan, Guizhou, and Xinjiang is higher than 1, indicating that the structure and effectiveness of pediatric medication in these regions continue to optimize and are in a state of progress. Among them, the average annual Malmquist index of Beijing, Shanxi, Jiangsu, Zhejiang, Anhui, Shandong, Guangdong, Sichuan, Jilin, and Fujian is higher than 1.05, indicating a significant growth optimization state. This is highly consistent with the reality of China's national conditions. Jiangsu, Zhejiang, Anhui, and Shandong are located in the economically developed East China region, which has a high level of infrastructure and excellent medical conditions, especially in the top 100 pediatric departments with a market share of over 70%. Beijing and Guangdong are the main economic and political regions in China, and their awareness of children's health care and medication level are also very excellent. Sichuan, Jilin, Fujian, and Shanxi are regional medical centers, with famous hospitals such as West China Hospital and Putian Medical Center located in these areas. Although the reputation of Putian hospitals varies, they provide a large amount of basic medical care and undertake a certain amount of workload in basic outpatient services, popularization of simple pediatric medication, and other aspects. From a yearly perspective, the Malmquist index performance of each region was relatively low from 2015 to 2016, with an average of 0.9843. However, from 2016 to 2019, the Malmquist index maintained stable growth. Due to the impact of the epidemic, there was a certain decline in 2019–2020, but in the era of epidemic and post epidemic, the Malmquist index for pediatric medication quickly recovered. Every crisis is also a challenge and adjustment. Major health events reshape the logic of pediatric medication, guiding refined, scientific, and standardized medication methods to serve pediatric patients. This led to the resurgence of the Malmquist index between 2020 and 2021. Further refining to each province, areas with excellent Malmquist index such as Beijing, Jiangsu, and Sichuan have input and output structures that are basically consistent with MDM matrix evaluation. According to the logic of each dominant factor and the overall goal, if the primary variable is not redundant as an input factor and significantly expands as an output result, it means that the Malmquist index performance in the region is better, that is, the efficacy of pediatric medication drugs is more outstanding. This further validates the effectiveness of MDM evaluation for pediatric medication and confirms the direction of standardized strategy development for pediatric medication.

## Discussion

This paper constructs a pediatric medication evaluation system based on the national conditions of developing countries, using MEM and lean management concepts; Through expert research and questionnaire collection, various levels and types of factors have been formed and their preliminary strong and weak relationships have been sorted out; By using the MEM calculation principle, the importance sequence of various factors for pediatric medication evaluation was finally obtained, which shows the proportion of various factors to the quality of pediatric medication. Based on the research results of this paper, it can serve hospitals, doctors, and parents' perception of pediatric medication, and capture the medication mechanisms and strategies under different goal orientations, greatly alleviating the medication conflicts caused by resource limitations in developing countries.

Considering the limitations of the study, this paper hopes that there will be more discussions in the follow-up study. First of all, as far as the object is concerned, this paper selects China, a typical representative of developing countries. However, due to the differences within developing countries, follow-up research can build an evaluation system based on the actual situation of other countries. And integrate the actual data of these countries to obtain more practical research results. Secondly, in terms of data, this paper selects the panel data from 2015 to 2021. Although this observation fully includes the pediatric construction in China. However, follow-up studies can further update the data and observe the performance of pediatric drug use in China after the epidemic. So, in the process of index collection and establishment of MDM matrix framework, this paper is carried out in the form of cross-sectional study. That is, the questionnaire data focus on regional feedback at a single time. The follow-up study can take the form of multi cycle and multi sampling to sort out the data, so as to further obtain the weight of pediatric drug MDM. Finally, due to the fact that there are differences in pediatric cognition within China, there may be potential medication bias. The follow-up research can enrich this issue based on samples and case studies. That is to discuss the development of pediatric drug use in China from the perspective of cognition and prejudice.

## Conclusions

Overall, the pre medication cycle is a critical period for pediatric medication evaluation, and multiple factors need to be coordinated during this time point. In the AF factor architecture, the importance of physician standardization is the highest, contributing about 1/4 of the evaluation weight. In the AF sub factor architecture, physician competence is the most important factor, contributing approximately 12.6% of the evaluation weight. Various national medical departments can further optimize the use of this evaluation model by considering factor variables with local characteristics and incorporating them into the evaluation system, in order to calculate factor variables that are more meaningful and important to the local area. Furthermore, when both parties (doctors and families) pursue medication time, parental cognition is the most important variable and also a reference variable. When emphasizing medication prices, the status of children is the reference variable, while the quality of doctors is the most important reference indicator. When medication efficacy is the guiding factor, the state of children is considered a reference variable, but hospital infrastructure is the most important reference variable. When emphasizing drug flexibility and addressing medication compatibility issues, it is necessary to use health records as a reference variable, but parental cognition is the most important indicator. When pursuing medication safety, physician competence serves as a reference variable and is also the most important feedback indicator. Medical institutions, personnel, and parents at all levels should adjust the weight of reference indicators in a timely manner according to their own goals in pediatric medication activities, in order to optimize and improve the effectiveness of pediatric medication.

Based on the evaluation framework of MDM and the real data of Chinese provinces, the MDM matrix is mapped into an input–output system. This paper analyzes the real efficacy of pediatric medication in each province from 2015 to 2021. From the results, it can be seen that the standardization of pediatric medication in China is significantly strengthening, and the efficiency of pediatric medication is showing an upward trend. Pediatric medication efficacy is relatively superior in medically developed or economically advanced regions, while pediatric medication is relatively redundant in economically or resource backward regions, and the overall trend of progress is relatively weak. From the perspective of input–output structure, the input–output ratio of pediatric medication efficacy leading regions is relatively consistent with the MDM matrix evaluation trend, that is, the scale of important output factors is relatively large, and the control of important input factors is relatively small. By simplifying and optimizing the structural ratio, pediatric medication maintains a good efficiency frontier, and the overall medication quality is greatly improved.

One contribution and goal of this paper is to help developing countries with limited medical conditions establish a pediatric medication evaluation system that is suitable for their national conditions. Although developing countries do not have a complete market and medication system for pediatric drugs, and lack effective innovation systems for pediatric drugs, there is even a shortage of some specific and specialized drugs. But within this framework, we can help users clearly indicate their medication goals and establish a pathway for key considerations towards medication goals. At the same time, we can also guide the medication subject to quickly establish a thinking paradigm. Help them quickly capture core variables in the complex outpatient process, avoiding unnecessary diagnostic and medication errors. Of course, in future research, we can incorporate more indicator factors and establish a multi-level feedback framework to form a more practical medication evaluation system. In addition, various factors represent multiple strategies for improving pediatric medication. During the post medication period, China is vigorously promoting the construction of medical cooperatives, which is reflected in the improvement of the medical system. Medical institutions can strengthen guidance and education for parents of children regarding common issues during and after medication. Establish a comprehensive medication follow-up system, especially to track and guide the medication use of discharged chronic disease children. Medical institutions should comprehensively utilize various methods such as community hospitals and medical publicity to carry out clinical rational drug use promotion for children, guide parents of children to establish scientific drug use concepts, improve safety medication awareness and children's medication compliance. Therefore, for other developing countries that use this evaluation framework, they can propose improvement plans for pediatric medication based on their own weak factors, strengthen and improve the scientific management system for pediatric medication at the grassroots level, and ultimately enhance the quality of pediatric healthcare nationwide.

## Supplementary Information

Supplementary Material 1.

## Data Availability

The data presented in this study are available on request from the corresponding author.
