# Supplementary material for: Modeling and scientific analysis of pediatric medication evaluation based on MDM-DEA-Malmquist model: construction of health management in pediatrics in developing countries
Source: Ital J Pediatr. 2025 Feb 7;51:29. doi: 10.1186/s13052-025-01893-0 (PMC11806530; doi:10.1186/s13052-025-01893-0)
Supplement: Supplementary file 1 — Supplementary Material 1. [file 13052_2025_1893_MOESM1_ESM.docx]

**Appendix A**

**Research on pediatric medication in developing countries**

Hello, thank you very much for your support in this research.

Since this study does not involve statistics and division of survey groups, basic items such as age and gender will not be statistically analyzed. But please ensure that you are engaged in or have a high understanding of pediatric medication practices before filling out. Our research targets mainly include three types of population, the first being doctors and experts specializing in pediatric medicine; The second type is parents who have children in their families and are mainly responsible for and understand their children's medication; The third category is professionals engaged in community or government healthcare work, or responsible for medical construction activities in universities.

As it is an on-site survey, you can consult the staff at any time for some unfamiliar questions:

The specific items are as follows

1.Please provide the goals for pediatric medication from the following options (a total of 100 points, please divide the scores into each item according to your evaluation approach)

Time: Price: Effect: Flexibility/Compatibility: Safety:

2. During the period before, during, and after pediatric medication, what do you think is the importance of the following:

(1: least important, 9: most important; if irrelevant, no value can be assigned)

Before medication

Time: Price: Effect: Flexibility/Compatibility: Safety:

During medication

Time: Price: Effect: Flexibility/Compatibility: Safety:

After medication

Time: Price: Effect: Flexibility/Compatibility: Safety:

In the cycles before, during, and after pediatric medication, do you think the following points indicate the importance of each cycle: (1: least important, 7: most important)

Before medication

Disease control: Parental attitude: Doctor's norms:

During medication

Parental behavior: Child feedback:

After medication

Medical cooperation: Feedback from parents:

4.Please provide your evaluation of the importance of the following factors in pediatric medication activities:

(1: least important, 7: most important)

Common diseases: Disease severity:

Child status: Parental cognition:

Hospital infrastructure: Doctor literacy: Doctor preferences:

Timeliness of medication: Control of medication:

Improvement rate of children: Drug resistance of children: Disease change rate: External environment:

Health records: Medication tracking:

Health habits: Medical network:

In addition, the above factors all point to the indicators of superior factors (shown in pictures by the staff). If you have any questions about their causal relationship, or if you have suggestions to add a relationship, you can tell us and point out scientific reasons. Thank you very much for your cooperation.
